# Desolvation and Development of Specific Hydrophobic Core Packing during Im7 Folding

**DOI:** 10.1016/j.jmb.2009.12.048

**Published:** 2010-03-12

**Authors:** Alice I. Bartlett, Sheena E. Radford

**Affiliations:** 1 Astbury Centre for Structural Molecular Biology, University of Leeds, Leeds LS2 9JT, UK; 2 Institute of Molecular and Cellular Biology, University of Leeds, Leeds LS2 9JT, UK

**Keywords:** U, unfolded ensemble, I, intermediate ensemble, N, native state, TS, transition state, *k*_*xy*_, the rate constant of folding/unfolding from *x* to *y*, *K*_*xy*_, the equilibrium constant between *x* and *y*, *M*_*xy*_, the denaturant dependence of the free energy between species *x* and *y*, *m*_*xy*_, the denaturant dependence of the natural logarithm of the rate constant *k_xy_*, NTL9, N-terminal domain of L9, EDTA, ethylenediaminetetraacetic acid, hydrophobic core, protein folding, Im7, solvation, phi-value analysis

## Abstract

Development of a tightly packed hydrophobic core drives the folding of water-soluble globular proteins and is a key determinant of protein stability. Despite this, there remains much to be learnt about how and when the hydrophobic core becomes desolvated and tightly packed during protein folding. We have used the bacterial immunity protein Im7 to examine the specificity of hydrophobic core packing during folding. This small, four-helix protein has previously been shown to fold *via* a compact three-helical intermediate state. Here, overpacking substitutions, in which residue side-chain size is increased, were used to examine the specificity and malleability of core packing in the folding intermediate and rate-limiting transition state. In parallel, polar groups were introduced into the Im7 hydrophobic core *via* Val→Thr or Phe→Tyr substitutions and used to determine the solvation status of core residues at different stages of folding. Over 30 Im7 variants were created allowing both series of substitutions to cover all regions of the protein structure. Φ-value analysis demonstrated that the major changes in Im7 core solvation occur prior to the population of the folding intermediate, with key regions involved in docking of the short helix III remaining solvent-exposed until after the rate-limiting transition state has been traversed. In contrast, overpacking core residues revealed that some regions of the native Im7 core are remarkably malleable to increases in side-chain volume. Overpacking residues in other regions of the Im7 core result in substantial (> 2.5 kJ mol^− 1^) destabilisation of the native structure or even prevents efficient folding to the native state. This study provides new insights into Im7 folding; demonstrating that whilst desolvation occurs early during folding, adoption of a specifically packed core is achieved only at the very last step in the folding mechanism.

## Introduction

Burial of nonpolar side-chains and the formation of a hydrophobic core drives the folding of water-soluble, globular proteins and is a key determinant of protein stability.^1–4^ Understanding how proteins fold requires the structural and energetic characterisation of every transition state (TS) and intermediate (I) ensemble populated, as well as the initial denatured ensemble and the final native state. The protein engineering approach,^5^ which enables the energetic contribution of individual side-chains to stabilising a TS or I state to be calculated and enumerated as Φ-values, has been applied to the folding of more than 20 small water-soluble proteins to date.^6^ Through use of this analysis, detailed views of folding pathways have emerged,^6^ which include insights into very early events in folding.^7,8^ For Φ-value analysis, nondisruptive deletion substitutions are typically created so as to minimise the likelihood of introducing new interactions that may alter the folding pathway or the native structure.^9^ Recent studies have also demonstrated that the creation of multiple substitutions at a single site can provide useful additional information about the interactions that stabilise transition states.^6^ In particular, increasing side-chain volume allows nonspecific hydrophobic burial *versus* specific packing interactions to be differentiated at different folding stages.^10–12^

A combination of hydrophobic side-chain truncations and substitutions with larger hydrophobic residues has been used to investigate the folding TS of the Fyn SH3 domain,^10^ the N-terminal domain of L9 (NTL9)^12^ and protein L.^13^ This approach has also been used to assess the malleability of core packing in a *de novo* designed protein.^14^ Analysis of more than 40 variants of the Fyn SH3 domain demonstrated that only three of the nine core residues examined are tightly packed in the TS ensemble with the other sites remaining more loosely packed at this stage of folding.^10^ A more recent study used over- and underpacked Fyn SH3 domain variants to demonstrate that a coarse-grained model that includes a simple consideration of hydrophobicity can accurately predict kinetically important non-native interactions that are transiently sampled during folding.^15^ Anil *et al.* have used both natural and unnatural amino acids to carry out a similar analysis of the TS of the N-terminal domain of L9 (NTL9).^12^ Their results revealed that for several NTL9 variants the folding rate increases with increased hydrophobicity, despite destabilisation of the native structure, consistent with the TS being less specifically packed than the native state.^12^ Despite these pioneering studies, however, systematic analyses of the effect of increasing side-chain size on core packing during folding remain rare. This is probably a result of the additional attention required to satisfy the assumptions of Φ-value analysis for variants containing such mutations.^6,9^ Nonetheless, provided that suitable care is taken to ensure the fidelity of the folding route and the native structure for the variants created, such studies can provide powerful insights into the formation of hydrophobic contacts during folding including whether they are specific and well packed or arise from general hydrophobic collapse.

In addition to overpacking mutations; substitutions such as Val→Thr and Phe→Tyr can be used to provide complementary information about the solvation status of core residues at different stages of folding.^16,17^ For this purpose, Val→Thr substitutions are ideal, as these residues are isosteric^18^ and destabilise the native protein *via* stabilisation of the unfolded ensemble.^19^ This approach has been used to investigate the solvation status of core residues during folding of the α-spectrin SH3 domain.^16^ The data indicated that the extent of solvation varies depending on the location of the different residues in the TS^16^ and provided a means of benchmarking theoretical studies that had successfully predicted the importance of hydrophobic core desolvation in overcoming the TS barrier.^16^ Pressure-jump relaxation techniques offer an alternative method to assess the hydration of protein TS following introduction of polar side chains into the protein core.^20,21^

**Fig. 1:**
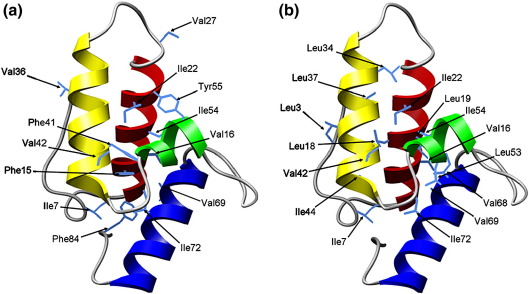
Ribbon diagrams of Im7 (PDB code 1AYI)^22^ showing core residues mutated to probe (a) solvation and (b) overpacking. Helix I is coloured red, helix II yellow, helix III green and helix IV blue. The figure was created using UCSF Chimera.^23^

Here, both side-chain overpacking and Val→Thr substitutions are used to examine the extent of desolvation and the development of specific hydrophobic packing interactions in the Im7 core during its folding transition. This small four-helix protein (Fig. 1) has previously been shown to fold *via* a compact on-pathway intermediate^24,25^ that is stabilised by both native and non-native interactions.^8,26^ The kinetic intermediate has been the subject of intense study.^27–30^ Previous Φ-value analysis has demonstrated that the intermediate is composed of helices I, II and IV, with the short third helix not forming or docking onto the developing structure until after the second, rate-limiting TS (TS2) has been traversed.^26^ Hydrogen-exchange NMR experiments later verified that the intermediate species contains extensive hydrogen-bonded secondary structure in helices I, II and IV.^28^ Manipulation of the Im7 folding landscape so that the intermediate became the dominant species populated at equilibrium^27^ enabled further biophysical characterisation of this species using spectroscopic methods and NMR.^27,29^ These studies demonstrated that the intermediate is highly helical but lacks a uniquely structured hydrophobic core.^27,29^ Despite these experiments and the prediction of details of the Im7 folding mechanism by combining mutational analysis and hydrogen exchange data with molecular dynamics simulations,^8,30^ key questions remain unresolved. These include the specificity of core packing and the extent of core desolvation at different stages of folding.

In this study, we present an analysis of the folding kinetics of a series of Im7 variants in which core Val or Phe residues were replaced with Thr or Tyr, respectively,^18^ to examine the extent of side-chain solvation in the intermediate ensemble and rate-limiting TS2. A second mutational analysis was performed in which the side-chains of core residues were increased in size, which enabled the specificity of core packing to be examined at the same distinct stages of folding. Together, these approaches provide new insights into hydrophobic core formation during Im7 folding, which include the extent to which specific side-chain packing interactions occur in non-native states and the degree to which desolvation affects the different stages of folding.

## Results

### Design of Im7 core mutants to probe solvation

Native Im7 adopts a simple four-helix structure in which the short third helix docks against the three long helices (I, II and IV)^22^ (Fig. 1). In order to probe the extent of side-chain desolvation in the I and rate-limiting TS2, suitable Val and Phe residues buried in the hydrophobic core were selected for analysis using prior knowledge of the Im7 folding mechanism.^8,26^ The solvent accessibility of different side-chains in the native structure was determined using DSSP.^31^ Where possible, substitutions were created at positions that had previously been mutated to Ala^26^ to enable comparison of the effect of truncation of a hydrophobic residue and introduction of a polar moiety at the same site. To enable all regions of the Im7 core to be probed, additional substitutions were made in which Ile residues were first substituted with Val and then with Thr. The Ile to Val substitution was then used as a pseudo wild-type to assess the effect of the subsequent Val to Thr substitution. Variants for which an initial Ile to Val substitution was created are denoted IV; for example, for Ile7 the Thr-containing variant is IV7T. In total, 13 Im7 variants were created to probe the solvent accessibility of the Im7 core as the protein folds. The residues substituted are highlighted in Fig. 1a and listed in Table 1. In addition to the 11 substitutions of core residues, Val to Thr substitutions were also made at two solvent-exposed positions as controls (Val27 and Val36). The introduction of Thr into core positions should stabilise the unfolded state thereby destabilising the native protein and any non-native species in which this side-chain is buried.^16^ However, if the side-chain is solvent-exposed in the folded structure, introduction of a Thr should not affect stability.^9,16^

### Folding and unfolding kinetics of Im7 solvation mutants

**Fig. 2:**
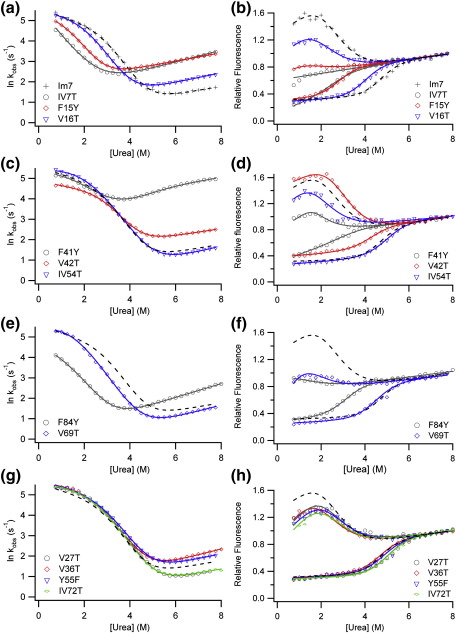
Folding and unfolding kinetics of the Im7 solvation variants. Chevron plots are shown in the left-hand panels (a, c, e and g); the corresponding initial and endpoint fluorescence data are shown in the right-hand panels (b, d, f and h). Variants created (a) in the N-terminal region and throughout helix I, (c) in helices II and III, and (e) in helix IV. Variants for which ΔΔ*G*°_UN_ was too small to calculate Φ-values are shown in (g). To facilitate comparison, the fit to the wild-type Im7 data is shown as a black dotted line in all plots. All data were acquired at pH 7.0, 10 °C, in the presence of 0.4 M Na_2_SO_4_, and fitted to a three-state on-pathway model (see Materials and Methods). The resulting kinetic and thermodynamic parameters are shown in Table 1.

The folding and unfolding kinetics of all 13 Im7 variants were measured by urea dilution experiments using conditions identical to those employed previously in studies of Im7 folding (pH 7, 10 °C, 0.4 M Na_2_SO_4_)^26^ (see Materials and Methods). With two exceptions (IV7T and IV22T), all of the Im7 variants fold *via* a three-state mechanism. This is demonstrated by the kinetic ‘rollover’ in the chevron plots of Im7 folding at low denaturant concentrations^32^ and the increase in fluorescence intensity in the burst phase (Fig. 2). For IV7T, the logarithm of the observed rate constant depends linearly on denaturant concentration over the entire range of urea concentrations studied, and there is no initial increase in fluorescence signal in the burst phase, indicating that the intermediate is substantially destabilised (Fig. 2a). The introduction of a Thr side chain at this site also causes substantial destabilisation of the native state (ΔΔ*G*°_UN_ = 14.8 ± 0.7 kJ mol^− 1^) (Fig. 2a and Table 1).

Truncation of hydrophobic side-chains is recommended as the ideal substitution for Φ-value analysis to avoid introducing new contacts or altering the native structure.^9^ The less conservative substitutions made here require careful analysis to assess whether alterations in the native structure have occurred. Reexamination of data from previous studies of Im7 folding^8^ indicates that the total *m* value (*M*_UN_ the denaturant dependence of ΔG°_UN_) for single point substitutions does not vary by greater than 10% from the value for wild-type Im7 (*M*_UN_ = 5.4 ± 0.1 kJ mol^− 1^ M^− 1^), with the *M*_UN_ value of most variants varying by less than 5%. Three of the variants analysed here (IV7T, IV54T and F41Y) show deviations greater than 10% (4.86 < *M*_UN_ < 5.94 kJ mol^− 1^ M^− 1^) (Table 1). F41Y displays curvature in both branches of the chevron plot (Fig. 2b and Table 1), consistent with I and N being similar in stability, such that both species are populated at equilibrium, possibly explaining the lower *M*_UN_ (4.6 ± 0.5 kJ mol^− 1^ M^− 1^) for this variant. One-dimensional (1D) ^1^H NMR spectra were recorded for both IV7T and IV54T and the relevant pseudo wild-type Im7 variants. All spectra displayed chemical shifts similar to those of wild-type Im7 and were therefore considered correctly folded (data not shown). The variants created in this series included two solvent-exposed positions, V27T and V36T. ΔΔ*G*°_UI_ and ΔΔ*G*°_UN_ for these variants are small (< 2.5 kJ mol^− 1^), as would be expected for introduction of Thr at a solvent-exposed position (Fig. 2g).

**Fig. 3:**
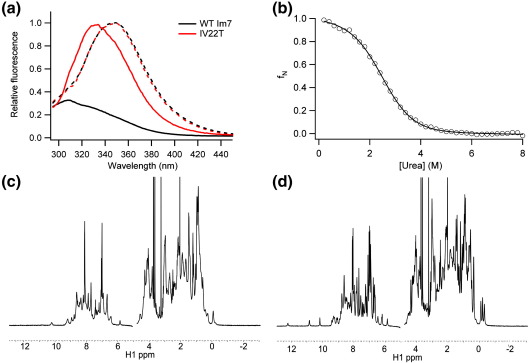
(a) Fluorescence emission spectra of IV22T (red) and wild-type Im7 (black) in 0 M (continuous line) or 8 M urea (dotted line). (b) Equilibrium denaturation curve of IV22T followed by tryptophan fluorescence. The data are fitted to a two-state transition. 1D ^1^H NMR spectra of (c) IV22T and (d) wild-type Im7. All data were acquired at pH 7.0, 10 °C, in the presence of 0.4 M Na_2_SO_4_.

Surprisingly, it was not possible to measure a chevron plot for the folding/unfolding kinetics of the variant IV22T. Ile22 is located in the C-terminal half of helix I (Fig. 1a). The IV22T variant displays unusual fluorescence properties compared with wild-type Im7 in that Trp fluorescence is no longer quenched in this variant (Fig. 3a). In wild-type Im7, His47 (located in the helix II–helix III loop) quenches the fluorescence of the sole Trp (Trp75, helix IV) in the native state,^33^ while the intermediate species is more fluorescent than both the native and unfolded states.^25,27^ Fitting of the equilibrium denaturation curve of IV22T, monitored using tryptophan fluorescence, yields Δ*G*°_UN_ = − 9.14 ± 0.59 kJ mol^− 1^ and *M*_UN_ = 3.72 ± 0.16 kJ mol^− 1^ M^− 1^ (Fig. 3b). The *M*_UN_ value for this variant is characteristic of the Im7 folding intermediate (for the trapped intermediate variant *M*_UN_ = 3.4 ± 0.09 kJ mol^− 1^ M^− 1^ and Δ*G*°_UN_ = − 10.1 ± 0.3 kJ mol^− 1^)^27^ as is its fluorescence λ_max_ (Fig. 3a). The data suggest the surprising result that substitution of Ile22 with Thr prevents folding to the native state. In accordance with this, the 1D ^1^H NMR spectrum of IV22T resembles that of the trapped intermediate^27^ with loss of the upfield-shifted methyl peaks characteristic of native Im7 (Fig. 3c and d). Introduction of the capacity to form hydrogen bonds with solvent by creating an Ile→Thr substitution at this position thus prevents folding to the native structure such that I is the dominant species at equilibrium. Interestingly, the substitution I22V has previously been shown to destabilise native Im7 by 8.9 kJ mol^− 1^, but to have no effect on the stability of the intermediate (ΔΔ*G*°_UI_ = 0.6 kJ mol^− 1^).^26^ Therefore, the Val→Thr substitution only needs to decrease the native-state stability by a further ∼ 3 kJ mol^− 1^ to tip the balance such that the folding intermediate becomes the predominant species at equilibrium.

### Analysis of Φ values for solvation variants

#### The N- and C-terminal regions of the Im7 core are buried early in folding

**Fig. 4:**
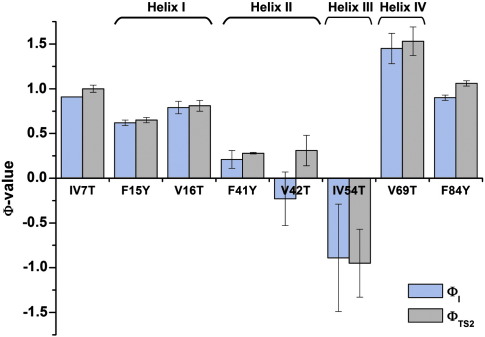
Histogram displaying Φ-values for the Im7 solvation variants. Blue bars depict Φ_I_; grey bars, Φ_TS2_. Variants for which ΔΔ*G*°_UN_ was too small to calculate Φ-values (< 2.5 kJ mol^− 1^) are not shown. Errors bars depict the errors on each Φ-value propagated mathematically from the errors determined on the fit parameters (see Materials and Methods).^8^ Data for all the solvation variants are shown in Table 1.

The six Val→Thr or Phe→Tyr variants that lie in the terminal regions of Im7 (IV7T, F84Y) or in helix I or IV (F15Y, V16T, V69T and IV72T) display high Φ-values (Φ_I_ > 0.62 and Φ_TS2_ > 0.65; Fig. 4 and Table 1). For all of these variants the Φ-values do not change significantly between I and TS2 (Fig. 4 and Table 1). These results indicate that the side-chains of these residues become buried from solvent early in folding, prior to formation of the intermediate species (which has a β_T_ value of ∼ 0.8 and forms on a submillisecond time scale).^8,25^ Final rearrangements and desolvation must occur in helix I after TS2 is traversed as residues in this helix have Φ_TS2_-values less than 1 (Table 1). The Φ-values for hydrophobic truncations created in the N-terminal region and helix I in previous studies are also less than 1.^8,26^

#### Core residues helices II and III remain solvent-exposed in I and the rate-limiting TS2

The most surprising results were obtained for variants created in the C-terminal half of helix II, F41Y and V42T (Figs. 1a and 4). Both these variants have low Φ_I_ values (Φ_I_ = 0.21 ± 0.10 for F41Y, − 0.23 ± 0.30 for V42T) and Φ_TS2_ values (Φ_TS2_ = 0.28 ± 0.01 and 0.31 ± 0.17 for F41Y and V42T, respectively) (Table 1 and Fig. 4). The data suggest that despite being at least partially docked in I and TS2 as indicated by hydrophobic deletion mutations in this region,^8,26^ the C-terminal region of helix II remains solvent-exposed until after TS2 has been traversed. Only a single mutation, IV54T, analysed in the short helix III resulted in a sufficient ΔΔ*G*°_UN_ (> 2.5 kJ mol^− 1^)^34,35^ for calculation of Φ-values. This variant results in negative Φ_I_ and Φ_TS2_ values (Table 1 and Fig. 4), suggestive of either non-native interactions in this region, changes in the unfolded state ensemble^36^ or subtle changes in the folding mechanism.^37^ Since the Φ-values obtained for hydrophobic truncations of this residue are low (Φ_I_ = 0.10 and Φ_TS2_ = 0.16 for I54V)^26^ non-native stabilising interactions in I and TS2 are unlikely, although no single explanation can be unequivocally ruled out.

While the chevron plot of IV54T is similar to that of wild-type Im7 (Fig. 2b), the Φ-values for this variant were calculated using the pseudo wild-type I54V as the reference state. I54V displays unusual folding/unfolding kinetics, with curvature in both arms of the chevron plot (data not shown) indicating that I and N are close to isoenergetic.^26^ I54V (Δ*G*°_UN_ of − 17.8 ± 1.70 kJ mol^− 1^) is significantly less stable than both IV54T and wild-type Im7 (Δ*G*°_UN_ = − 23.1 ± 0.40 and − 25.2 ± 0.65 kJ mol^− 1^, respectively). The loss of the δ-methyl of Ile54 by substitution with Val thus results in significant destabilisation of Im7. However, the subsequent Val→Thr substitution compensates for this by stabilising the protein, consistent with this region of the protein remaining at least partially solvent-exposed in the native state.

#### Im7 solvation and truncation variants provide complementary information

**Fig. 5:**
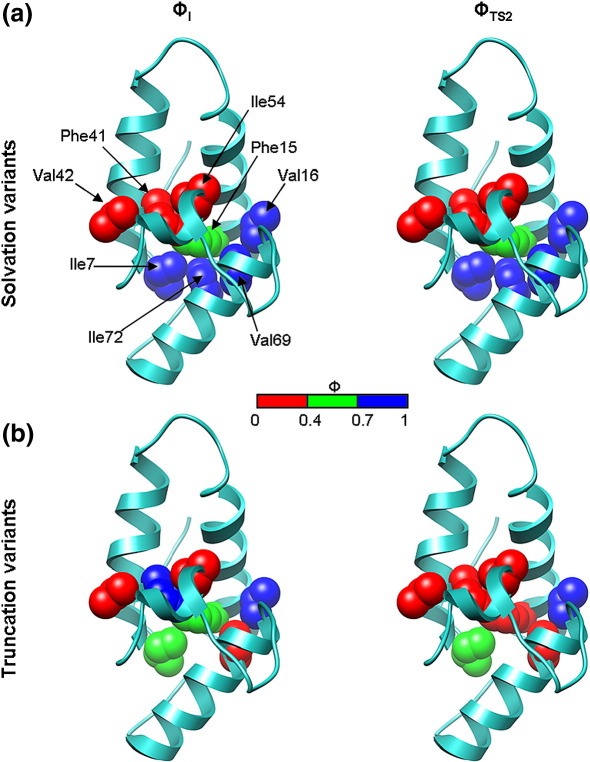
Comparison of the Φ-values measured for I and TS2 of Im7 using (a) solvation (Val→Thr, Phe→Tyr) or (b) truncation (Val→Ala, etc.) substitutions. Φ-values for the truncation variants were taken from Capaldi *et al.*^26^ Residues mutated are coloured according to their Φ-value (Table 1).^26^ Red indicates Φ < 0.4, green 0.4 < Φ < 0.7, blue Φ > 0.7. Data for Φ_I_ are shown in the left-hand panel and Φ_TS2_ on the right.

Comparison of the data from the original Φ-value analysis of Im7, in which hydrophobic side-chains were truncated,^26^ with the data provided by the V→T and F→Y substitutions presented here, provides new insights into hydrophobic core development during Im7 folding (Fig. 5). The combined data sets indicate that the regions of the protein that remain solvent exposed until late in folding (Φ_I_ and Φ_TS2_ < 0.4, side-chains coloured red in Fig. 5a) also have low Φ-values when the same residue is truncated (Fig. 5b). Phe41 is the only exception: the substitution F41L has a high Φ_I_-value, but a low Φ_TS2_ (∼ 1 and ∼ 0.27, respectively),^26^ indicating the intermediate is stabilised by non-native interactions in this region.^26^ By comparison, the solvation Φ-values for F41Y are low for both of these states (Φ_I_ = 0.21 ± 0.10, Φ_TS2_ = 0.28 ± 0.01) (Fig. 5a). Together, the data indicate that whilst the transition between I and TS2 involves reorganisation of side-chain packing, this occurs without large changes in overall compaction of the molecule (β_T_ = 0.8 and 0.9 for I and TS2, respectively; Table 1) or solvation status of core residues.

### Design of Im7 variants to examine the specificity of core packing during folding

To probe the specificity and malleability of the core packing in I and TS2 a series of overpacked Im7 variants were created in which side-chain size was increased for residues in the hydrophobic core. As for the solvation variants described above, previous knowledge of Im7 folding mechanism,^26^ in conjunction with analysis of the solvent accessibility of side-chains in native Im7 using DSSP,^31^ was used in selecting sites for mutation. Where possible, residue size was increased gradually, maintaining the gross chemical characteristics of the side-chain, in the series Val→Ile→Phe. In addition, a number of Leu→Phe substitutions were created to enable all regions of the Im7 structure to be sampled (Fig. 1b). In total, 18 overpacking variants were created spanning all four helices of the native Im7 structure. For these variants, large destabilisations indicate that core packing in the vicinity of the mutation is specific and likely to involve the tight interdigitation of side-chains. By contrast, small changes in stability indicate that packing in the vicinity of the mutation is tolerant to increases in side-chain volume, presumably because the structure is malleable at that site. By analysing the folding kinetics of the overpacked Im7 variants in this manner, it is thus possible to compare the specificity and malleability of packing interactions in I and TS2 with those in the final native structure.

### Analysis of the structure of the overpacked Im7 variants

**Fig. 6:**
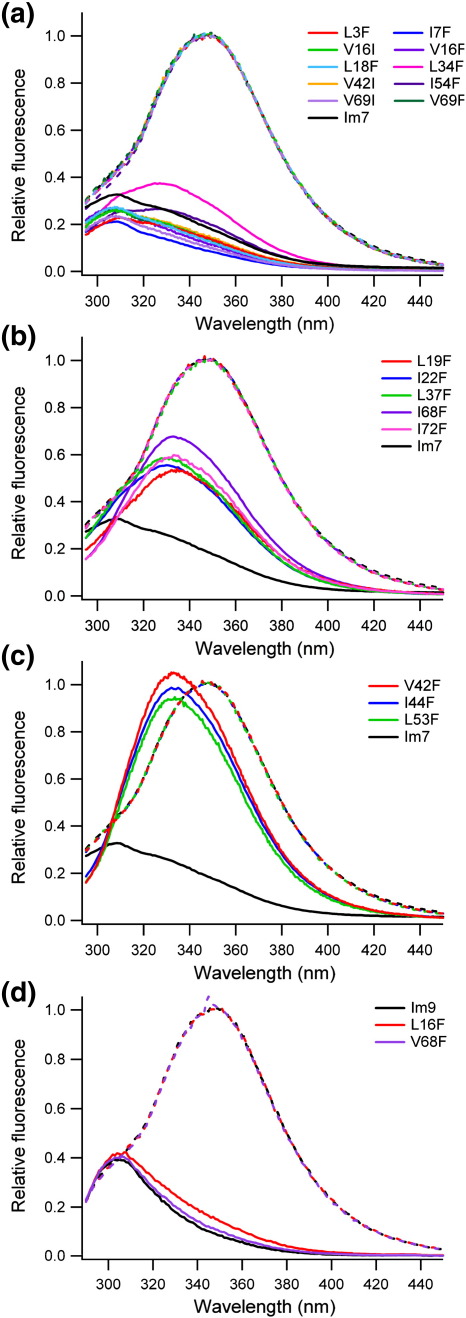
Fluorescence emission spectra of the overpacked Im7 variants in 0 M (continuous line) or 8 M (dotted line) urea. (a) Overpacked variants with fluorescence properties resembling that of the native wild-type protein; (b) variants that differ slightly in their fluorescence intensity compared with native wild-type Im7; (c) variants that have a fluorescence emission spectrum in 0 M urea that resembles that of the intermediate species (highly fluorescent, λ_max_ ∼ 335 nm).^27^ (d) Fluorescence emission spectra of Im9 and the overpacked Im9 variants created in this study.

Before analysis of the folding and unfolding kinetics of the overpacked variants, the effect of increasing the size of a buried side-chain on the native structure of Im7 was assessed. Tryptophan fluorescence emission spectra were recorded for each variant, exploiting the unique sensitivity of this technique to detect and identify the unfolded, intermediate and native states of Im7^26,27,33^ (Fig. 6). Ten of the variants displayed fluorescence emission spectra similar to that of wild-type Im7 (Fig. 6a). A further five variants displayed spectra with slightly increased fluorescence intensity relative to the native state of wild-type Im7 (Fig. 6b). Three of the variants, V42F, I44F and L53F, displayed fluorescence emission spectra that were more reminiscent of the intermediate species, with a high fluorescence intensity and a λ_max_ close to 335 nm,^27^ suggesting perturbation of the structural core, at least in the vicinity of Trp75 (Fig. 6c).

To gain further insight into the structural changes caused by introduction of these point substitutions, 1D ^1^H NMR spectra were acquired for all variants (Supplementary Figs. 1–3). The 10 variants whose native fluorescent emission spectra were similar to that of the wild-type protein (Fig. 6a) gave rise to 1D ^1^H NMR spectra that closely resemble that of wild-type Im7 (Supplementary Fig. 1), confirming they have adopted the native fold. Three of the five variants whose fluorescence spectra deviated slightly from that of wild-type Im7 (L19F, I22F, L37F) (Fig. 6b) also displayed NMR spectra similar to that of the native wild-type protein (Supplementary Fig. 2). The spectra of I68F and I72F, by contrast, showed significant broadening compared with wild-type Im7, suggesting that these variants may be improperly folded or may populate both the native and intermediate ensembles in intermediate exchange on the NMR time scale (Supplementary Fig. 2). The three variants with highly intense fluorescent emission spectra (V42F, I44F and L53F; Fig. 6c) display broad peaks in the 1D ^1^H NMR spectra and lack the upfield-shifted methyl peaks characteristic of native wild-type Im7 (Supplementary Fig. 3). The spectra of these variants resemble the spectrum of the trapped-intermediate variant of Im7.^27^ For these variants, both the fluorescence emission spectra and the 1D ^1^H NMR spectra suggest that a partially folded species is highly, or even predominantly, populated at equilibrium under the conditions employed.

In order to characterise the species populated at equilibrium by V42F, I44F and L53F in more detail, equilibrium denaturation experiments were carried out using far-UV CD as the structural probe (Supplementary Fig. 4 and Table 2). The results revealed that all three variants are significantly destabilised compared with wild-type Im7 (ΔΔ*G*°_UN_ > ∼ 6 kJ mol^− 1^, Table 2). The values of Δ*G*°_UN_ and *M*_UN_ determined for L53F closely resemble those of the trapped intermediate variant (L53AI54A) for which Δ*G*°_UN_ = − 11.1 ± 1.3 kJ mol^− 1^ and *M*_UN_ = 3.5 ± 0.35 kJ mol^− 1^ M^− 1^.^27^ The *M*_UN_ values recorded for V42F and I44F are also lower than those of the native state of wild-type Im7 (*M*_UN_ = 4.8 ± 0.1 kJ mol^− 1^ M^− 1^),^24^ which suggests that these variants do not adopt a native-like structure (Table 2). The results are consistent with these variants co-populating the native and intermediate species at equilibrium or, in the case of L53F, the intermediate becoming the predominant species at equilibrium. The data illustrate that the hydrophobic core of the native protein is tightly packed and nonmalleable in the vicinity of these residues, such that increasing side-chain volume causes substantial destabilisation of the native fold. Val42 and Ile44 are located in the C-terminal half of helix II, while Leu53 is situated in the short third helix (Fig. 1b). By contrast, the intermediate is not destabilised by increasing side-chain size at these sites, indicating that this region of the core is more malleable/loosely packed at this stage of folding.

### Folding and unfolding kinetics of the overpacked Im7 core variants

**Fig. 7:**
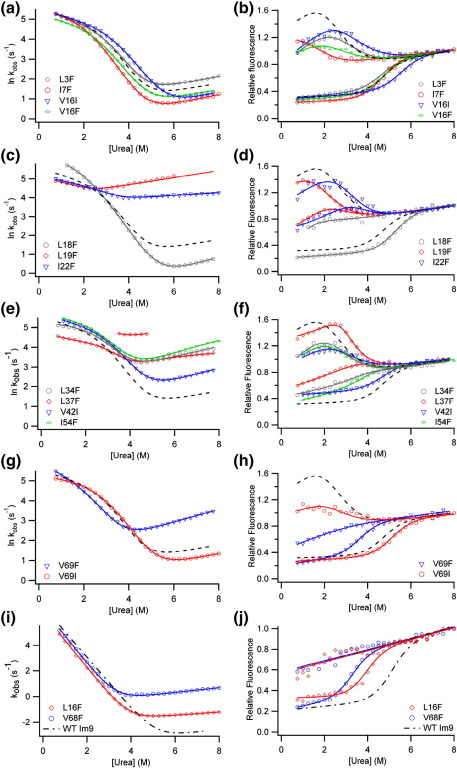
Folding and unfolding kinetics of overpacked Im7 and Im9 variants. Chevron plots are shown in the left-hand panels (a, c, e, g and i); the corresponding initial and endpoint fluorescence data are shown in the right-hand panels (b, d, f, h and j). Variants created in (a) the N-terminal region and the N-terminal half of helix I, (c) the C-terminal half of helix I, (e) helices II and III and (g) helix IV. To facilitate comparison, the fit to the wild-type Im7 data is shown as a black dotted line in all plots. All data were acquired at pH 7.0, 10 °C, in the presence of 0.4 M Na_2_SO_4_ and fitted to a three-state on-pathway model (see Materials and Methods). (i) Chevron plots for overpacked Im9 variants and (j) corresponding initial and endpoint fluorescence data. Im9 data were acquired at pH 7.0, 10 °C, and fitted to a two-state model (see Materials and Methods).

The folding and unfolding kinetics of the overpacked Im7 variants are shown in Fig. 7. All the overpacked Im7 variants, except I68F and I72F, exhibit kinetic rollovers in the refolding branch of their chevron plots, indicating that, like wild-type Im7,^25^ these variants also fold *via* a populated intermediate. Examination of the *M*_UN_ values for these variants provides further evidence that they adopt a native structure, at least in terms of protein surface area buried from solvent^38^ (as for the solvation variants, the total *m* value, *M*_UN_, is within ± 10% of the wild-type value; Table 3).

**Fig. 8:**
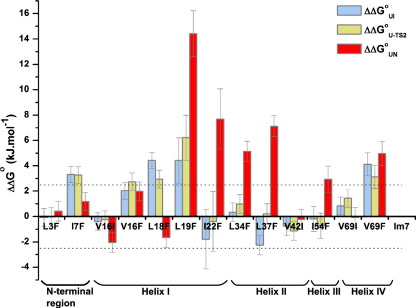
Histogram displaying ΔΔ*G*°_*xy*_ values for different states of the overpacked Im7 variants. Dotted black lines indicate ΔΔ*G*°_*xy*_ ± 2.5 kJ mol^− 1^.

The stability of the I and N states for the 13 overpacked Im7 variants that fold to the native structure, determined by fitting the chevron plots in Fig. 7 to a three-state mechanism, are shown in Fig. 8. These data show that a substantial number (7/13) of the overpacked Im7 variants are only marginally destabilised compared with the wild-type protein (ΔΔ*G*°_UN_ < 2.5 kJ mol^− 1^, the minimum value required for accurate determination of a Φ-value)^34,35^ (Table 3 and Fig. 8). Indeed, all of the variants containing Val→Ile substitutions (V16I, V42I and V69I) had little effect on native-state stability regardless of their position in the Im7 core.

#### Impact of overpacking core residues at the N-terminus and in helices I and II

Four substitutions involving substantial increases in side-chain volume (Val→Phe, Leu/Ile→Phe) gave rise to values of ΔΔ*G*°_UN_ < ± 2.5 kJ mol^− 1^ (Fig. 8). These four variants are located close to the Im7 N-terminus (L3F, I7F) and in the N-terminal portion of helix I (V16F, L18F) (Fig. 1b). Two of the variants created in this region, V16I and L18F (Δ*G*°_UN_ = − 27.2 ± 0.44 and −26.8 ± 0.43 kJ mol^− 1^, respectively), are in fact slightly stabilised compared with wild-type Im7 (Δ*G*°_UN_ = − 25.19 ± 0.65 kJ mol^− 1^) (Table 3). The data suggest that the native Im7 core is highly malleable, at least in the vicinity of these residues. ΔΔ*G*°_UI_ and ΔΔ*G*°_U-TS2_ for residues in this region are largely of similar magnitude to ΔΔ*G*°_UN_. However, for I7F and L18F, I and TS2 are more destabilised than the native structure, suggesting that packing of side chains in the vicinity of these residues may involve tighter, more specific organisation in I (and TS2) than in the native structure. Regardless of the structural interpretations, the very different responses of I and TS2 to mutation compared with N highlights the known importance of non-native interactions in stabilising these species.^8,26^ By contrast with residues in the N-terminal region of helix I, ΔΔ*G*°_UI_ and ΔΔ*G*°_U-TS2_ are generally substantially smaller than ΔΔ*G*°_UN_ for residues in the C-terminal half of helix I (I22F) and in helix II (L34F, L37F). This indicates that in these regions, packing in these transient species is better able to accommodate the introduction of bulky side chains than the native structure (Fig. 8 and Table 3).

Overpacking residues in the C-terminal half of helix I (probed using L19F and I22F) and the N-terminal half of helix II (L34F, L37F) results in Im7 variants destabilised by > 5 kJ mol^− 1^ (Fig. 8 and Table 3), indicating the native core is more specifically (tightly) packed in this region. This destabilisation predominantly arises from large increases in *k*_NI_ (5- to 30-fold compared with the value for wild-type Im7) (Fig. 7c and e). By contrast, analysis of ΔΔ*G*°_UI_ and ΔΔ*G*°_UTS2_ revealed much smaller destabilisation (Fig. 8), consistent with the transient ensembles populated during folding remaining less specifically packed at these sites. The L19F substitution is of particular interest, as this variant attains the native structure despite being destabilised by > 14 kJ mol^− 1^ (Fig. 8 and Table 3). For this variant, I and TS2 are destabilised to a similar extent as N by the introduction of a Phe residue at this position (Fig. 8). By contrast, those variants that fail to adopt the native structure (V42F, Ile44 and L53F) destabilise the native structure but have a much smaller effect on the stability of I and TS2, thus tipping the landscape such that I becomes partially or predominantly populated at equilibrium. A number of mutations introduced in the C-terminal half of helix I and the N-terminal half of II hairpin have different effects on the stability of the intermediate and the native state. Thus, I22F and L37F destabilise the native structure while stabilising the intermediate ensemble (Fig. 8 and Table 3). In contrast, the L18F substitution stabilises the native structure but destabilises both the intermediate and TS2 ensembles (Fig. 8 and Table 3). Together, these results demonstrate that the intermediate is not simply a loosely packed hydrophobically collapsed state,^8,27^ but involves the tight and specific interdigitation of side chains, at least at some sites.

#### Overpacking residues in helices III and IV has dramatic consequences for Im7 folding

Of the two overpacking substitutions (L53F, I54F) created in the short helix III, only I54F resulted in a sequence able to attain the native structure (Fig. 1b). Like the L53F substitution, which failed to fold to the correct native structure and predominantly populated the intermediate at equilibrium, the I54F variant does not alter the stability of the intermediate state or TS2 (Fig. 8 and Table 3). By contrast with the L53F substitution, the I54F variant results in only a small destabilisation of the native state (Δ*G*°_UN_ = − 22.3 kJ mol^− 1^; Table 3), indicating that core packing in this region is exquisitely sensitive to side-chain orientation in the native structure.

Four variants were created to examine packing of core residue side-chains in helix IV; however the folding/unfolding kinetics could only be measured for variants created at a single site (Val69). The V69F substitution results in similar destabilisations of I, TS2 and N (Fig. 8 and Table 3), which suggests that the specificity of core packing in this region is highly developed throughout the folding transition.

The chevron plots for I68F and I72F could not be fitted to the three-state on-pathway model used for the other Im7 variants (see Materials and Methods), since *k*_obs_ for these variants has virtually no denaturant dependence, resulting in chevron plots that resemble flat lines (data not shown). Far-UV CD equilibrium denaturation experiments (Supplementary Fig. 4) demonstrated that *M*_UN_ for these variants is low (*M*_UN_ = 3.4 ± 0.2 kJ mol^− 1^ M^− 1^ for I68F and 3.2 ± 0.2 kJ mol^− 1^ M^− 1^ for I72F; Table 2) compared with that of the wild-type protein (4.8 ± 0.1 kJ mol^− 1^)^24^. Combined with 1D ^1^H NMR spectra (Supplementary Fig. 2), the data suggest that these variants are either in dynamic equilibrium between the native and intermediate ensembles or predominantly populate a partially folded structure at equilibrium. In the native structure, Ile68 and Ile72 are located on the same face of helix IV as Trp75. Molecular dynamics simulations using Φ-values^8^ and equilibrium hydrogen-exchange protection factors^30^ as restraints suggest that Trp75 makes substantial non-native interactions with aromatic residues in the intermediate ensemble. It is perhaps not surprising that I68F and I72F disrupt folding and appear to populate multiple conformers at equilibrium.

#### Comparing the malleability of the Im7 core with that of the homologous protein Im9

The ability of Im7 to tolerate large changes in side-chain size is surprising given that hydrophobic truncations at the same sites typically result in significant ΔΔ*G*°_UN_ values.^26^ To identify whether this behaviour is specific to Im7, a number of overpacked variants of the homologous immunity protein, Im9, were created. Im7 and Im9 share 60% sequence identity yet fold *via* different kinetic mechanisms at neutral pH.^24,39–41^ Overpacking substitutions were therefore created at Leu16 and Val68 in Im9, the equivalent to positions Val16 and Val69 in Im7. The fluorescence emission spectra of the Im9 variants overlay with that of wild-type Im9 (Fig. 6d) indicating that these substitutions do not alter the native structure. The ΔΔ*G*°_UN_ values for these variants in Im9 are also large (ΔΔG°_UN_ = 5.67 ± 0.60 and 8.19 ± 0.59 kJ mol^− 1^ for L16F and V68F, respectively) (Fig. 7i and j and Supplementary Table 1) and much greater than the effects observed for the equivalent substitutions in Im7 (ΔΔ°G_UN_ = 1.97 ± 0.75 kJ mol^− 1^ for V16F and 4.96 ± 0.95 kJ mol^− 1^ for V69F; Fig. 8 and Table 3). Im7 thus appears to be unusual in its ability to tolerate overpacking substitutions, at least in the regions of its hydrophobic core in the vicinity of Val16 (helix I) and Val69 (helix IV).

## Discussion

**Fig. 9:**
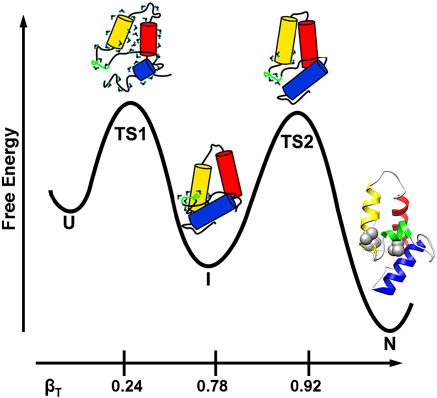
Schematic of Im7 folding. Helices are coloured as in Fig. 1. The β_T_ value for TS1 was taken from Ref. 8; values for other states are taken from the wild-type Im7 data in Table 1. Water molecules are shown as black and green (^) symbols. While the core of TS1 is assumed to remain highly solvated,^8^ the data presented here demonstrate the substantial desolvation occurs as I forms. The final steps in folding involve the docking of core residues (Val42, Ile44 and L53) in the C-terminal portion of helix II and in helix III, which serve to lock the protein into its native structure. These key residues are highlighted on the ribbon diagram of native Im7 (PDB code 1AYI).^22^ Overpacking these positions thus prevents efficient folding to the native state such that I becomes partially or wholly populated at equilibrium.

Although Im7 folding has been studied in detail using a wide range of approaches,^8,26–30,33,41^ several key questions regarding the extent of desolvation and specificity of packing in the hydrophobic core at different stages in folding remained unresolved. Here, the creation of multiple point substitutions throughout the Im7 core has enabled the solvation status and specificity of packing interactions for core residues to be determined during the different stages of Im7 folding. The data revealed that many side-chains involved in the native Im7 hydrophobic core become largely buried from solvent early in the folding process, as demonstrated by the high Φ_I_ and Φ_TS2_ values recorded for the Val→Thr and Phe→Tyr substitutions (Fig. 9). However, residues in the C-terminal portion of helix II and all those studied in helix III display low Φ_I_ and Φ_TS2_ values for Val→Thr and Phe→Tyr substitutions, suggesting that these regions of the polypeptide chain remain solvated until the very final stages of folding. Previous Φ-value analyses using hydrophobic truncation variants^8,26^ demonstrated that residues in helices I, II and IV have intermediate Φ_I_ and Φ_TS2_ values, while those in helix III have values that are close to zero (− 0.02 to 0.16) in these states.^26^ Together with the results presented here, a picture emerges in which I and TS2 possess a nascent core that is largely desolvated, but remains fluidly packed throughout folding (Fig. 9). These data are consistent with predictions using molecular dynamics simulations, which also suggested that core desolvation occurs by the time the intermediate species forms.^8^ Previous studies using a protein engineering approach to examine core solvation in α-spectrin SH3^16^ and azurin^17^ have also shown that core residues are solvated to different extents in the TS ensemble, suggesting that differential solvation of different regions of the polypeptide chain is a common feature of non-native species formed during folding.

Overpacking core residues in regions of Im7 that remain solvent-exposed until after TS2 has been traversed has dramatic and previously unanticipated consequences for folding. The substitutions V42F, I44F (helix II) and L53F (helix III) dramatically destabilise N, whilst having relatively little effect on the stability of I, with the result that both I and N are populated at equilibrium. Core packing interactions in this region of Im7, despite remaining solvent-exposed until late in folding, appear to be crucial for stabilising the native structure and hence are intolerant to substitution. Previous results examining the impact of increasing the size of core side chains on the stability of T4 lysozyme concluded that there may be a limit to the loss of stability that can result from induced strain within native protein structures.^42^ Liu *et al*. suggest that increasing strain in the native structure above a certain threshold is released by changes in structure.^42^ The results observed here for the V42F, I44F and L53F variants of Im7 concur with these conclusions. Overpacking residues in the C-terminal half of helix I and throughout helix II result in large positive ΔΔ*G*°_UN_ values, indicating the native Im7 is also tightly packed in this region. However, over a third of the overpacking substitutions created had limited consequences for Im7 folding and resulted in small ΔΔ*G*°_UN_ values (< ± 2.5 kJ mol^− 1^; Fig. 8). Thus, despite being highly organised, a substantial portion of the native Im7 core is able to tolerate the introduction of bulky hydrophobic residues. This behaviour appears to be unique to Im7 since it is not shared by its homologue Im9. It is not possible to determine whether the malleability displayed by regions of the Im7 core is especially unusual for a small helical protein, since previous studies using overpacking substitutions to examine folding kinetics and stability have been confined to date to all beta and mixed alpha/beta proteins.^10,12^

Using a computational method to quantify the degree of frustration in localised interactions in protein molecules,^43^ Sutto *et al.* identified a number of highly frustrated interactions in native Im7 in the loop between helices I and II and in the helical regions adjacent to this loop.^44^ This analysis suggested that frustrated interactions that arise from sequence requirements for function (colicin binding) result in the formation of non-native interactions in the folding intermediate and lead to the characteristic rugged landscape for Im7 folding.^44^ It is not possible, however, to differentiate between the presence of frustrated interactions and malleable core packing using mutational analysis, since both could result in a limited destabilisation upon increasing side-chain volume. Regardless of the structural interpretation of the magnitude of destabilisation observed here for different states in Im7 folding, the observation that several residues exhibit different responses to overpacking in I and N (Fig. 8 and Table 3) adds further weight to the body of evidence that Im7 folds *via* an intermediate stabilised by both native and non-native interactions. Here, this is revealed by the finding that I and N are stabilised by different packing interactions in this region of the developing hydrophobic core, confirming previous suggestions from both experiment^8,26^ and simulation^8,30,44^ for folding *via* transient non-native interactions involving residues important for Im7 function.

In recent years, a number of studies have demonstrated the advantage of creating multiple different amino acid substitutions at a single site for interrogating hydrophobic core development during folding .^6,10,15^ The two sets of Im7 variants created here to probe both solvation status and packing specificity in the hydrophobic core provide information complementary to previous Φ-value analysis involving hydrophobic truncation mutations.^8,26^ Both sets of data presented here point to the importance of the C-terminal region of helix II and helix III in stabilising the native structure. The final steps in folding involves the desolvation and docking of residues in these regions into the hydrophobic core effectively locking Im7 into its native structure (Fig. 9). Although helix III was known not to dock onto the developing Im7 structure until late in folding,^8,26^ the specific details of the mechanism by which the native hydrophobic core is formed could only be elucidated by using multiple variants to probe different aspects of structure formation during folding. An earlier experimental study demonstrated that the formation of the kinetic intermediate is an essential step in Im7 folding and that intermediate formation does not arise simply as a consequence of the low helical propensity of the short (six residue) helix III.^45^ MD simulations restrained by experimental Φ-values^8^ and hydrogen-exchange protection factors^30^ highlighted a number of non-native interactions involving core residues in the C-terminal half of helix II and helix III as important determinants of the stability of the intermediate. Here we have shown the final steps to form native Im7 involve desolvation coupled with development of exquisitely tight packing of residues that form the C-terminal region of helix II and helix III. The overpacking substitutions thereby revealed key lynchpin residues in the Im7 sequences that are required to lock the protein into a uniquely structured and functional native state.

## Materials and Methods

### Mutagenesis and protein purification

Mutagenesis was carried out using the Quikchange site-directed mutagenesis kit (Stratagene) with the Im7 gene in pTrc-99A as the template.^41^ All proteins were expressed with a hexahistidine tag and purified as described,^41^ with the following modifications: anion-exchange purification was carried out using Source15Q resin (GE Healthcare), the protein was loaded onto the column in 50 mM sodium phosphate buffer (pH 6.0), washed with two column volumes of buffer and then eluted with a gradient of 0–0.65 M NaCl in the same buffer. Proteins were > 95% pure as determined by SDS-PAGE.

### Kinetic experiments

All kinetic experiments were performed using an Applied-Photophysics SX1.8MV stopped-flow fluorimeter. The temperature was held constant at 10 °C (± 0.1 °C) using a Neslab circulating water bath system. Experiments were performed in buffered solutions containing 50 mM sodium phosphate (pH 7.0), 400 mM Na_2_SO_4_ and 1 mM ethylenediaminetetraacetic acid (EDTA). Experiments on Im9 variants were performed in 50 mM sodium phosphate (pH 7.0), 2 mM DTT and 1 mM EDTA. Refolding experiments were performed by 1:10 dilution of ∼ 50 μM protein in buffer containing 8 M urea into buffered solutions with final urea concentrations in the range 0.75–8.0 M. Unfolding experiments were measured in the same way but the initial protein solution did not contain urea. The final urea concentration ranged from 3.0 to 8.0 M for unfolding experiments. At each urea concentration at least seven kinetic traces were obtained, averaged and fitted to a single- or double-exponential function using the manufacturer's software. Initial and final fluorescence signals were determined from the fit to the kinetic refolding reactions. Buffer blanks were subtracted from both the initial and final fluorescence signals, which were then normalised to the fluorescence signal of the 7.75 M urea sample.

**Scheme 1:**
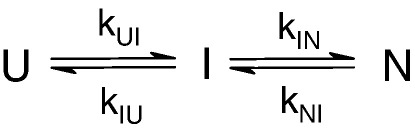
Reaction scheme for a three-state on-pathway folding mechanism.

The observed rate constants, endpoint and initial fluorescence signals for each variant were fitted to the analytical solution for an on-pathway three-state model (Scheme 1) using Igor Pro 6.0 (Wavemetrics) as described in Ref. 8. According to Scheme 1, *k*_UI_ and *m*_UI_ were fixed to the values obtained for wild-type Im7 in continuous-flow mixing experiments (*k*_UI_ = 1574 s^− 1^, *m*_UI_ = 1.23 kJ mol^− 1^ M^− 1^),^8^ while the stability of the kinetic intermediate was determined by allowing *k*_IU_ to vary.^40^ The data for the L37F variant, for which *k*_UI_ could be measured directly, was fitted as described in Ref. 8 constraining the total *m* value (*M*_UN_) to the range determined in equilibrium denaturation studies (*M*_UN_ 4.6–5.2 kJ mol^− 1^ M^− 1^). Φ-values for I and TS2 were calculated from the microscopic rate constants according to Eqs. (1) and (2).

(1) $$\Phi_{\text{I}}=\Delta \Delta G_{\text{UI}}^{\text{mut−WT}}/\Delta \Delta G_{\text{UN}}^{\text{mut−WT}}$$

(2) $$\Phi_{\text{TS2}}=\Delta \Delta G_{\text{UI}}^{\text{mut−WT}}-RT\ln\left(\frac{k_{\text{IN}}^{\text{mut}}}{k_{\text{IN}}^{\text{WT}}}\right)/\Delta \Delta G_{\text{UN}}^{\text{mut−WT}}$$

**Scheme 2:**
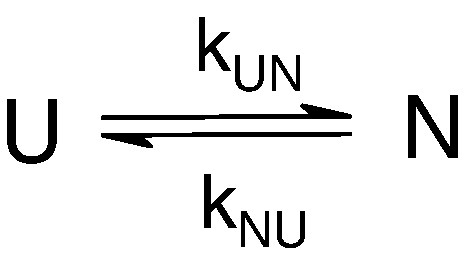
Reaction scheme for a two-state folding transition.

Errors were propagated mathematically from the errors determined on the fit parameters.^8^ The observed rate constants, endpoint and initial fluorescence signals for Im9 variants and the IV7T Im7 variant were simultaneously fit to a two-state model (Scheme 2) using Igor Pro 6.0 (Wavemetrics).

### Fluorescence spectra

Fluorescence emission spectra of each of the overpacked Im7 variants were measured using a Photon Technology International Fluorimeter (Ford, West Sussex, UK). For spectra of native and denatured Im7 variants, each protein was dissolved in buffer [50 mM sodium phosphate (pH 7.0), 400 mM Na_2_SO_4_ and 1 mM EDTA] containing 0 M (native) or 8 M (denatured) urea (protein concentration ∼ 5 μM) and incubated at 10 °C overnight. Excitation slit widths were set to 2 nm; emission slit widths were adjusted for protein concentration. Each spectrum was recorded from 270 nm to 450 nm in 1 nm increments, using an excitation wavelength of 280 nm. Spectra of all denatured states were assumed to have the same maximum intensity at 350 nm. The spectra of each native protein were normalised to the intensity of their respective denatured state, allowing direct comparison of the fluorescence intensity between variants.

### Equilibrium denaturation curves

Equilibrium denaturation curves were monitored using either fluorescence (IV22T) or far-UV CD (V42F, I44F, L53F, I68F and I72F). In both cases, solutions contained 0–8 M urea in 0.2 M increments in buffer [50 mM sodium phosphate (pH 7.0), 400 mM Na_2_SO_4_ and 1 mM EDTA]. Final protein concentrations were 5 μM for fluorescence experiments and 18 μM for CD. All measurements were at 10 °C and samples were preincubated overnight in a Neslab circulating water bath at 10 °C (± 0.1 °C). Fluorescence data were measured using a Photon Technology International Fluorimeter, with a 1-cm path-length cuvette. Fluorescence was excited at 280 nm and emission at 350 nm was monitored and averaged over 1 min. CD equilibrium denaturation curves were measured on a Jasco J715 CD spectropolarimeter, using a 1-mm path-length cuvette. The CD signal at 225 nm was monitored and averaged over 1 min using a response time of 1 s and bandwidth of 1 nm. The average signal as a function of denaturant concentration was fitted to an equation defining a two-state transition according to Eq. (3) using Igor Pro 6.0 (Wavemetrics):

(3) $${\text{S}}_{\text{obs}}=\frac{\left[\left(a\left[\text{D}\right]+b\right)\exp\left(\Delta {G^{\circ }}_{\text{UN}}^{{\text{H}}_{2}\text{O}}-M_{\text{UN}}\left[\text{D}\right]/RT+\left(c\left[\text{D}\right]+d\right)\right)\right]}{\left(1+\exp\left(\Delta {G^{\circ }}_{\text{UN}}^{{\text{H}}_{2}\text{O}}-M_{\text{UN}}\left[\text{D}\right]/RT\right)\right)}$$

where *a* and *c* are the signals of the native and denatured states, respectively, in the absence of denaturant, and *b* and *d* are the denaturant dependence of the signal of the native and denatured states, respectively.^24^ For equilibrium denaturation curves monitored using fluorescence, the observed signal (*S*_obs_) was then converted into the fraction of native protein (*f*_N_) according to the equation:

(4) $$f_{\text{N}}=\frac{S_{\text{obs}}-\left(c\left[\text{D}\right]+d\right)}{\left(a\left[\text{D}\right]+b\right)-\left(c\left[\text{D}\right]+d\right)}$$

where *a*, *b*, *c* and *d* are as specified above for Eq. (3) and [D] is the denaturant concentration.

### NMR

1D ^1^H NMR spectra were recorded at 10 °C on a Varian Inova 500-MHz spectrometer, averaging 256 transients. Samples contained approximately 0.5 mM protein dissolved in 50 mM sodium phosphate (pH 7.0), 400 mM Na_2_SO_4_ and 1 mM EDTA containing 10% ^2^H_2_O. Spectra were plotted using NMRPipe.^46^

## Figures and Tables

**Table 1:** Parameters determined from the best fit of the folding/unfolding kinetics of Im7 solvation variants

Variant	Position	*K*_UI_ (*M*_UI_)	*k*_IN_ (*m*_IN_)	*k*_NI_ (*m*_NI_)	Δ*G*°_UI_ (kJ mol^− 1^)	Δ*G*°_UN_ (kJ mol^− 1^)	Φ_I_	Φ_TS2_	β_I_	β_TS2_
IV7T^a^	N term		*k*_UN_ 279.4 ± 10.69 (m_UN_ 3.27 ± 0.06)	k_NU_ 3.40 ± 0.14 (m_NU_ 0.68 ± 0.01)	− 1.84 ± 1.22^a^	− 10.38 ± 0.13	0.73^b^	1.00 ± 0.04		0.83 ± 0.005
F15Y	Helix I	13.9 ± 3.02 (4.25 ± 0.18)	220.2 ± 14.77 (0.64 ± 0.34)	5.95 ± 0.12 (0.48 ± 0.01)	− 6.19 ± 0.51	− 14.69 ± 0.54	0.62 ± 0.03	0.65 ± 0.03	0.79 ± 0.05	0.91 ± 0.02
V16T	Helix I	52.9 ± 7.30 (4.18 ± 0.06)	244.5 ± 9.57 (0.49 ± 0.11	1.79 ± 0.08 (0.55 ± 0.02)	− 9.34 ± 0.32	− 20.90 ± 0.35	0.79 ± 0.07	0.81 ± 0.06	0.80 ± 0.02	0.90 ± 0.01
V27T	Loop 1	137.7 ± 16.49 (4.17 ± 0.05)	250.2 ± 8.60 (0.37 ± 0.07)	0.69 ± 0.08 (0.51 ± 0.04)	− 11.59 ± 0.28	− 25.46 ± 0.39	ND	ND	0.83 ± 0.01	0.90 ± 0.01
V36T	Helix II	77.6 ± 11.29 (3.99 ± 0.06)	276.6 ± 11.63 (0.32 ± 0.1)	1.05 ± 0.08 (0.68 ± 0.03)	− 10.24 ± 0.34	− 23.35 ± 0.40	ND	ND	0.80 ± 0.02	0.86 ± 0.01
F41Y	Helix II	80.0 ± 46.95 (3.37 ± 0.46)	186.1 ± 15.74 (0.84 ± 0.17)	40.43 ± 9.83 (0.40 ± 0.07)	− 10.31 ± 1.38	− 13.90 ± 1.51	0.21 ± 0.10	0.28 ± 0.01	0.73 ± 0.04	0.91 ± 0.03
V42T	Helix II	300.2 ± 62.79 (4.27 ± 0.10)	125.0 ± 3.30 (0.47 ± 0.05)	3.19 ± 0.19 (0.41 ± 0.02)	− 13.42 ± 0.49	− 22.06 ± 0.52	− 0.23 ± 0.30	0.31 ± 0.17	0.83 ± 0.01	0.92 ± 0.01
IV54T^a^	Helix III	53.43 ± 6.21 (4.06 ± 0.08)	229.5 ± 11.90 (0.03 ± 0.13)	0.66 ± 0.07 (0.62 ± 0.04)	− 9.36 ± 0.27	− 23.13 ± 0.40	− 0.89 ± 0.60	− 0.95 ± 0.38	0.86 ± 0.02	0.87 ± 0.01
Y55F	Helix III	133.4 ± 22.41 (3.89 ± 0.06)	286.7 ± 9.77 (0.58 ± 0.07)	1.28 ± 0.12 (0.55 ± 0.03)	− 11.51 ± 0.40	− 24.25 ± 0.46	ND	ND	0.78 ± 0.01	0.89 ± 0.01
V69T	Helix IV	20.01 ± 2.08 (4.51 ± 0.23)	225.8 ± 21.52 (0.42 ± 0.29)	0.53 ± 0.05 (0.68 ± 0.03)	− 7.05 ± 0.24	− 21.30 ± 0.40	1.45 ± 0.17	1.53 ± 0.16	0.80 ± 0.04	0.88 ± 0.01
IV72T^a^	Helix IV	110.9 ± 13.70 (4.00 ± 0.05)	273.7 ± 9.66 (0.48 ± 0.08)	0.79 ± 0.07 (0.47 ± 0.03)	− 11.08 ± 0.29	− 24.84 ± 0.36	ND	ND	0.85 ± 0.04	0.88 ± 0.01
F84Y	C term	7.55 ± 2.04 (3.25 ± 0.18)	114.4 ± 5.64 (1.02 ± 0.35)	1.01 ± 0.02 (0.80 ± 0.01)	− 4.76 ± 0.64	− 15.88 ± 0.65	0.90 ± 0.03	1.06 ± 0.03	0.64 ± 0.05	0.84 ± 0.03
Im7		222.2 ± 55.56 (4.19 ± 0.10)	253.9 ± 11.46 (0.75 ± 0.09)	1.26 ± 0.14 (0.45 ± 0.04)	− 12.71 ± 0.59	− 25.19 ± 0.65	—	—	0.78 ± 0.01	0.92 ± 0.01

Φ-values were calculated from *k*_IU_, *k*_IN_ and *k*_NI_. For IV7T, the data were fitted to equations describing a two-state model of folding. β_T_ values, which describe the solvent exposure of I or TS relative to the native state, were calculated from kinetic *m* values according to the equation: $\beta_{\text{T}}=\frac{m_{U-x}}{m_{U-x}+m_{x-N}}$ The units of *k* are s^− 1^; units of *M*/*m* are kJ mol^− 1^ M^− 1^.

a Indicates proteins for which Φ-values have been calculated using a pseudo wild-type (the corresponding I→V variant).

b The chevron plot for this variant was fitted to equations describing apparent two-state folding and the maximum possible Δ*G*°_UI_ estimated by modelling the minimum Δ*G*°_UI_ required for refolding branch rollover to be observed (∼ 1 kJ mol^− 1^). The difference between the Δ*G*°_UN_ obtained for IV7T from equilibrium denaturation (Δ*G*°_UN_ − 12.22 ± 1.21 kJ mol^− 1^, data not shown) and kinetic experiments (Δ*G*°_UN_ − 10.38 ± 0.13 kJ mol^− 1^) provides another means of estimating Δ*G*°_UI_. With this approach, a Δ*G*°_UI_ of − 1.84 ± 1.22 kJ mol^− 1^ was determined for the IV7T variant. ND Φ-values were not calculated for these variants as ΔΔ*G*°_UN_ was too small (< 2.5 kJ mol^− 1^).^34,35^

**Table 2:** Equilibrium stabilities and *m* values determined from equilibrium denaturation experiments monitored using far-UV CD (data shown in SI Figure 4)

Variant	Position	Δ*G*°_UN_ (kJ mol^− 1^)	*M*_UN_ (kJ mol^− 1^ M^− 1^)
V42F	Helix II	− 19.2 ± 0.8	4.2 ± 0.2
I44F	Helix II	− 16.2 ± 1.9	4.4 ± 0.5
L53F	Helix III	− 13.5 ± 1.2	3.5 ± 0.3
I68F	Helix IV	− 9.3 ± 0.9	3.4 ± 0.2
I72F	Helix IV	− 9.2 ± 0.8	3.2 ± 0.2

**Table 3:** Parameters determined from the best fit of the folding/unfolding kinetics of the overpacked Im7 variants

Variant	Position	*K*_UI_ (*M*_UI_)	*k*_IN_ (*m*_IN_)	*k*_NI_ (*m*_NI_)	Δ*G*°_UI_ (kJ mol^− 1^)	Δ*G*°_UN_ (kJ mol^− 1^)	Φ_I_	Φ_TS2_	β_I_	β_TS2_
L3F	N term	229 ± 38.9 (3.98 ± 0.07)	250.7 ± 6.11 (0.86 ± 0.05)	1.54 ± 0.09 (0.5 ± 0.02)	− 12.8 ± 0.40	− 24.8 ± 0.42	ND	ND	0.75 ± 0.01	0.91 ± 0.01
I7F	N term	54.3 ± 5.8 (3.90 ± 0.07)	258.3 ± 9.52 (0.70 ± 0.10)	0.52 ± 0.03 (0.55 ± 0.02)	− 9.4 ± 0.25	− 24.0 ± 0.30	ND	ND	0.76 ± 0.01	0.89 ± 0.01
V16I	Helix I	262.8 ± 37.02 (3.7 ± 0.05)	238.4 ± 5.6 (0.69 ± 0.04)	0.59 ± 0.07 (0.55 ± 0.04)	− 13.1 ± 0.33	− 27.2 ± 0.44	ND	ND	0.75 ± 0.01	0.89 ± 0.01
V16F	Helix I	93.7 ± 13.3 (3.78 ± 0.05)	187.7 ± 5.6 (0.67 ± 0.07)	0.91 ± 0.06 (0.46 ± 0.02)	− 10.7 ± 0.33	− 23.2 ± 0.38	ND	ND	0.77 ± 0.01	0.91 ± 0.01
L18F	Helix I	34.0 ± 2.9 (4.17 ± 0.22)	474.1 ± 63.1 (0.18 ± 0.27)	0.18 ± 0.02 (0.75 ± 0.03)	− 8.3 ± 0.20	− 26.8 ± 0.43	ND	ND	0.82 ± 0.04	0.85 ± 0.01
L19F	Helix I	34.0 ± 24.2 (4.14 ± 0.38)	118.3 ± 7.4 (0.73 ± 0.34)	41.2 ± 1.36 (0.56 ± 0.02)	− 8.3 ± 1.7	− 10.8 ± 1.7	0.31 ± 0.09	0.43 ± 0.07	0.76 ± 0.05	0.90 ± 0.02
I22F^a^	Helix I	471.8 ± 456.0 (4.5 ± 0.6)	141.3 ± 9.0 (0.97 ± 0.14)	39.4 ± 2.0 (0.18 ± 0.02)	− 14.5 ± 2.3	− 17.5 ± 2.3	− 0.23 ± 0.37	− 0.05 ± 0.32	0.80 ± 0.03	0.97 ± 0.02
L34F	Helix II	192.4 ± 34.2 (4.2 ± 0.1)	193.5 ± 8.6 (0.42 ± 0.08)	7.39 ± 0.51 (0.60 ± 0.03)	− 12.4 ± 0.4	− 20.1 ± 0.5	0.1 ± 0.1	0.2 ± 0.1	0.81 ± 0.01	0.88 ± 0.01
L37F	Helix II	579.1 ± 125 (4.22 ± 0.14)	89.0 ± 2.3 (0.78 ± 0.025)	23.9 ± 0.74 (0.17 ± 0.01)	− 15.0 ± 0.51	− 18.1 ± 0.52	0.01 ± 0.07	0.27 ± 0.05	0.82 ± 0.01	0.97 ± 0.01
V42I	Helix II	306.5 ± 55.1 (4.01 ± 0.07)	298.1 ± 8.65 (0.75 ± 0.05)	1.85 ± 0.10 (0.68 ± 0.02)	− 13.5 ± 0.42	− 25.4 ± 0.5	ND	ND	0.74 ± 0.01	0.87 ± 0.01
I54F	Helix III	240.5 ± 81.0 (4.12 ± 0.15)	320.5 ± 14.7 (0.71 ± 0.10)	6.03 ± 0.27 (0.75 ± 0.02)	− 12.9 ± 0.79	22.3 ± 0.81	− 0.06 ± 0.36	− 0.25 ± 0.42	0.74 ± 0.02	0.87 ± 0.01
V69I	Helix IV	156.1 ± 23.8 (3.97 ± 0.06)	196.9 ± 7.2 (0.45 ± 0.08)	0.67 ± 0.07 (0.51 ± 0.03)	− 11.9 ± 0.36	− 25.3 ± 0.44	ND	ND	0.80 ± 0.01	0.90 ± 0.01
V69F	Helix IV	38.6 ± 11.2 (3.51 ± 0.09)	387.9 ± 14.4 (1.28 ± 0.15)	2.76 ± 0.09 (0.75 ± 0.01)	− 8.6 ± 0.68	− 20.2 ± 0.69	0.83 ± 0.06	0.63 ± 0.08	0.63 ± 0.02	0.87 ± 0.01
Im7		222.2 ± 55.56 (4.19 ± 0.10)	253.9 ± 11.46 (0.75 ± 0.09)	1.26 ± 0.14 (0.45 ± 0.04)	− 12.71 ± 0.59	− 25.19 ± 0.65	—	—	0.78 ± 0.01	0.92 ± 0.01

Φ-values were calculated from *k*_IU_, *k*_IN_ and *k*_NI_. The units of *k* are s^− 1^; units of *M*/*m* are kJ mol^− 1^ M^− 1^. ND Φ-values were not calculated for these variants as ΔΔ*G*°_UN_ was too small (< 2.5 kJ mol^− 1^).^34^

a The data for I22F were modelled to a three-state on-pathway mechanism and the best model parameters were used as initial values for fitting. The large errors on parameters determined for this variant result from its poorly defined chevron plot.

## References

[1] Kellis J.T., Nyberg K., Sali D., Fersht A.R. (1988). Contribution of hydrophobic interactions to protein stability. Nature.

[2] Baldwin R.L. (2007). Energetics of protein folding. J. Mol. Biol..

[3] Dill K.A., Ozkan S.B., Shell M.S., Weikl T.R. (2008). The protein folding problem. Annu. Rev. Biophys..

[4] Ventura S., Serrano L. (2004). Designing proteins from the inside out. Proteins.

[5] Matouschek A., Kellis J.T., Serrano L., Fersht A.R. (1989). Mapping the transition state and pathway of protein folding by protein engineering. Nature.

[6] Zarrine-Afsar A., Davidson A.R. (2004). The analysis of protein folding kinetic data produced in protein engineering experiments. Methods.

[7] Calosci N., Chi C.N., Richter B., Camilloni C., Engström Å., Eklund L. (2008). Comparison of successive transition states for folding reveals alternative early folding pathways of two homologous proteins. Proc. Natl Acad. Sci. USA.

[8] Friel C.T., Smith D.A., Vendruscolo M., Gsponer J., Radford S.E. (2009). The mechanism of formation of a folding intermediate reveals the competition between functional and kinetic evolutionary constraints. Nat. Struct. Mol. Biol..

[9] Fersht A.R., Matouschek A., Serrano L. (1992). The folding of an enzyme: I. Theory of protein engineering analysis of stability and pathway of protein folding. J. Mol. Biol..

[10] Northey, J. G. B, Di Nardo, A.A., Davidson A.R. (2002). Hydrophobic core packing in the SH3 domain folding transition state. Nat. Struct. Biol..

[11] Bertagna A.M., Barrick D. (2004). Nonspecific hydrophobic interactions stabilize an equilibrium intermediate of apomyoglobin at a key position within the AGH region. Proc. Natl Acad. Sci. USA.

[12] Anil B., Sato S., Cho J.-H., Raleigh D.P. (2005). Fine structure analysis of a protein folding transition state; distinguishing between hydrophobic stabilization and specific packing. J. Mol. Biol..

[13] Kim D.E., Fisher C., Baker D. (2000). A breakdown of symmetry in the folding transition state of protein L. J. Mol. Biol..

[14] Walsh S.T.R., Sukharev V.I., Betz S.F., Vekshin N.L., DeGrado W.F. (2001). Hydrophobic core malleability of a de novo designed three-helix bundle protein. J. Mol. Biol..

[15] Zarrine-Afsar A., Wallin S., Neculai A.M., Neudecker P., Howell P.L., Davidson A.R., Chan H.S. (2008). Theoretical and experimental demonstration of the importance of specific nonnative interactions in protein folding. Proc. Natl Acad. Sci. USA.

[16] Fernandez-Escamilla A.M., Cheung M.S., Vega M.C., Wilmanns M., Onuchic J.N., Serrano L. (2004). Solvation in protein folding analysis: combination of theoretical and experimental approaches. Proc. Natl Acad. Sci. USA.

[17] Wilson C.J., Apiyo D., Wittung-Stafshede P. (2006). Solvation of the folding-transition state in *Pseudomonas aeruginosa* azurin is modulated by metal. Protein Sci..

[18] Chothia C. (1976). The nature of the accessible and buried surfaces in proteins. J. Mol. Biol..

[19] Serrano L., Kellis J.T., Cann P., Matouschek A., Fersht A.R. (1992). The folding of an enzyme: II. Substrucutre of barnase and the contribution of different interactions to protein stability. J. Mol. Biol..

[20] Brun L., Isom D.G., Velu P., Garcia-Moreno B., Royer C.A. (2006). Hydration of the folding transition state ensemble of a protein. Biochemistry.

[21] Mitra L., Hata K., Kono R., Maeno A., Isom D., Rouget J.-B. (2007). *V*_*i*_-value analysis: a pressure-based method for mapping the folding transition state ensemble of proteins. J. Am. Chem. Soc..

[22] Dennis C.A., Videler H., Pauptit R.A., Wallis R., James R., Moore G.R., Kleanthous C. (1998). A structural comparison of the colicin immunity proteins Im7 and Im9 gives new insights into the molecular determinants of immunity-protein specificity. Biochem. J..

[23] Eric F.P., Thomas D.G., Conrad C.H., Gregory S.C., Daniel M.G., Elaine C.M., Thomas E.F. (2004). UCSF Chimera—a visualization system for exploratory research and analysis. J. Computational Chem..

[24] Ferguson N., Capaldi A.P., James R., Kleanthous C., Radford S.E. (1999). Rapid folding with and without populated intermediates in the homologous four-helix proteins Im7 and Im9. J. Mol. Biol..

[25] Capaldi A.P., Shastry M.C.R., Kleanthous C., Roder H., Radford S.E. (2001). Ultrarapid mixing experiments reveal that Im7 folds via an on-pathway intermediate. Nat. Struct. Mol. Biol..

[26] Capaldi A.P., Kleanthous C., Radford S.E. (2002). Im7 folding mechanism: misfolding on a path to the native state. Nat. Struct. Mol. Biol..

[27] Spence G.R., Capaldi A.P., Radford S.E. (2004). Trapping the on-pathway folding intermediate of Im7 at equilibrium. J. Mol. Biol..

[28] Gorski S.A., Le Duff C.S., Capaldi A.P., Kalverda A.P., Beddard G.S., Moore G.R., Radford S.E. (2004). Equilibrium hydrogen exchange reveals extensive hydrogen bonded secondary structure in the on-pathway intermediate of Im7. J. Mol. Biol..

[29] Whittaker S.B.M., Spence G.R., Gunter Grossmann J., Radford S.E., Moore G.R. (2007). NMR analysis of the conformational properties of the trapped on-pathway folding intermediate of the bacterial immunity protein Im7. J. Mol. Biol..

[30] Gsponer J., Hopearuoho H., Whittaker S.B.M., Spence G.R., Moore G.R., Paci E. (2006). Determination of an ensemble of structures representing the intermediate state of the bacterial immunity protein Im7. Proc. Natl Acad. Sci. USA.

[31] Kabsch W., Sander C. (1983). Dictionary of protein secondary structure: pattern recognition of hydrogen-bonded and geometrical features. Biopolymers.

[32] Brockwell D.J., Radford S.E. (2007). Intermediates: ubiquitous species on folding energy landscapes?. Curr. Opin. Struct. Biol..

[33] Rodriguez-Mendieta I.R., Spence G.R., Gell C., Radford S.E., Smith D.A. (2005). Ultraviolet resonance Raman studies reveal the environment of tryptophan and tyrosine residues in the native and partially folded states of the E colicin-binding immunity protein Im7. Biochemistry.

[34] Fersht A.R., Sato S. (2004). Φ-value analysis and the nature of protein-folding transition states. Proc. Natl Acad. Sci. USA.

[35] Sánchez I.E., Kiefhaber T. (2003). Origin of unusual φ-values in protein folding: evidence against specific nucleation sites. J. Mol. Biol..

[36] Cho J.-H., Raleigh D.P. (2006). Denatured state effects and the origin of nonclassical values in protein folding. J. Am. Chem. Soc..

[37] de los Rios M.A., Daneshi M., Plaxco K.W. (2005). Experimental investigation of the frequency and substitution dependence of negative Φ-values in two-state proteins. Biochemistry.

[38] Myers J.K., Pace C.N., Scholtz J.M. (1995). Denaturant *m* values and heat capacity changes: Relation to changes in accessible surface areas of protein unfolding. Protein Sci..

[39] Friel C.T., Beddard G.S., Radford S.E. (2004). Switching two-state to three-state kinetics in the helical protein Im9 via the optimisation of stabilising non-native interactions by design. J. Mol. Biol..

[40] Morton V.L., Friel C.T., Allen L.R., Paci E., Radford S.E. (2007). The effect of increasing the stability of non-native interactions on the folding landscape of the bacterial immunity protein Im9. J. Mol. Biol..

[41] Gorski S.A., Capaldi A.P., Kleanthous C., Radford S.E. (2001). Acidic conditions stabilise intermediates populated during the folding of Im7 and Im9. J. Mol. Biol..

[42] Liu R., Baase W.A., Matthews B.W. (2000). The introduction of strain and its effects on the structure and stability of T4 lysozyme. J. Mol. Biol..

[43] Ferreiro D.U., Hegler J.A., Komives E.A., Wolynes P.G. (2007). Localizing frustration in native proteins and protein assemblies. Proc. Natl Acad. Sci. USA.

[44] Sutto L., Latzer J., Hegler J.A., Ferreiro D.U., Wolynes P.G. (2007). Consequences of localized frustration for the folding mechanism of the IM7 protein. Proc. Natl Acad. Sci. USA.

[45] Knowling S.E., Figueiredo A.M., Whittaker S.B.M., Moore G.R., Radford S.E. (2009). Amino acid insertion reveals a necessary three-helical intermediate in the folding pathway of the colicin E7 immunity protein Im7. J. Mol. Biol..

[46] Delaglio F., Grzesiek S., Vuister G.W., Zhu G., Pfeifer J., Bax A. (1995). NMRPipe: a multidimensional spectral processing system based on UNIX pipes. J. Biomol. NMR.

