# Supplementary material for: Desolvation and Development of Specific Hydrophobic Core Packing during Im7 Folding
Source: J Mol Biol. 2010 Mar 12;396(5):1329–45. doi: 10.1016/j.jmb.2009.12.048 (PMC2833379; doi:10.1016/j.jmb.2009.12.048)
Supplement: Supplementary data [file mmc1.doc]

**Supplementary Information**

**Figure Legends**

**SI Figure 1**

1D ^1^H NMR spectra of the over-packed variants of Im7 whose native fluorescence emission spectra resemble that of the wild-type protein (Figure 6a).

**SI Figure 2**

1D ^1^H NMR spectra of the over-packed variants of Im7 whose native fluorescence emission spectra differ slightly from wild-type Im7, but lack the fluorescence characteristic of the intermediate species (Figure 6b).

**SI Figure 3**

1D ^1^H NMR spectra of the over-packed variants of Im7 whose native fluorescence emission spectra resemble that of the trapped intermediate variant of Im7 (Figure 6c).

**SI Figure 4**

Equilibrium denaturation curves of (a) V42F, (b) I44F, (c) L53F, (d) I68F and (e) I72F monitored using Far-UV CD. All data were acquired at pH 7.0, 10°C, in the presence of 0.4M Na_2_SO_4_ and fitted to a two state transition (see Methods). Equilibrium stabilities and M_UN_-values determined from the fits are shown in Table 2.

**SI Table 1**

Parameters determined from the best fit of the folding/unfolding kinetics of the over-packed Im9 variants. The data were acquired at pH 7.0, 10°C, in the absence of Na_2_SO_4_. The units of k are s^-1^, units of M/m are kJ mol^-1^.M^-1^.

**SI Figure 1**

**
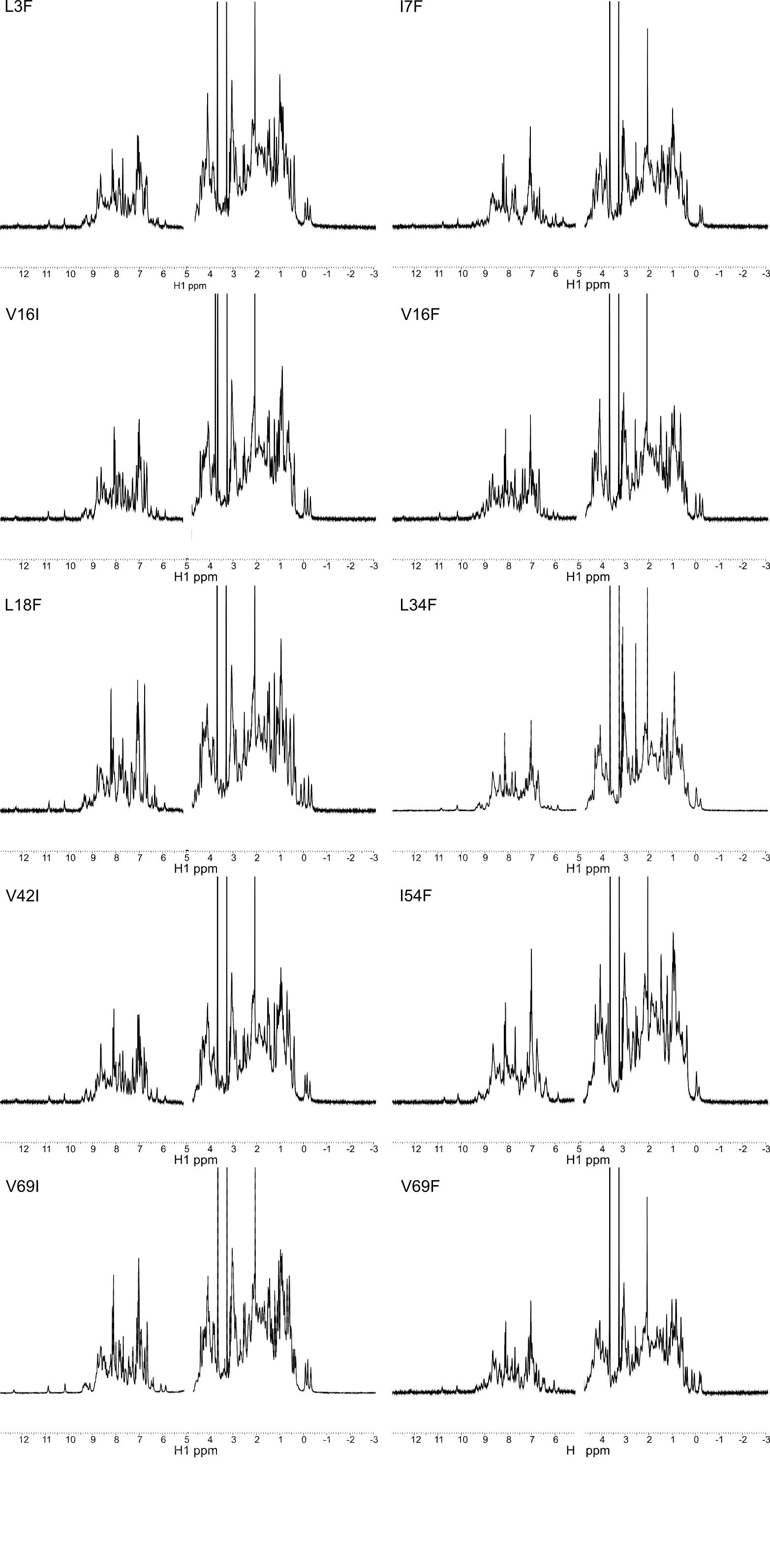
**

**SI Figure 2**

**
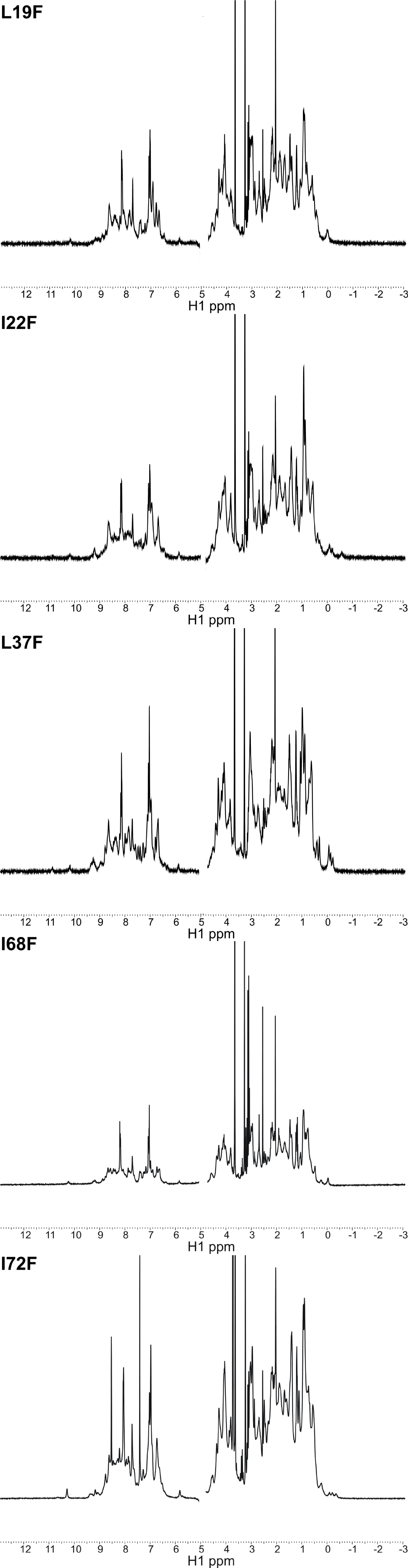
**

**SI Figure 3**

**
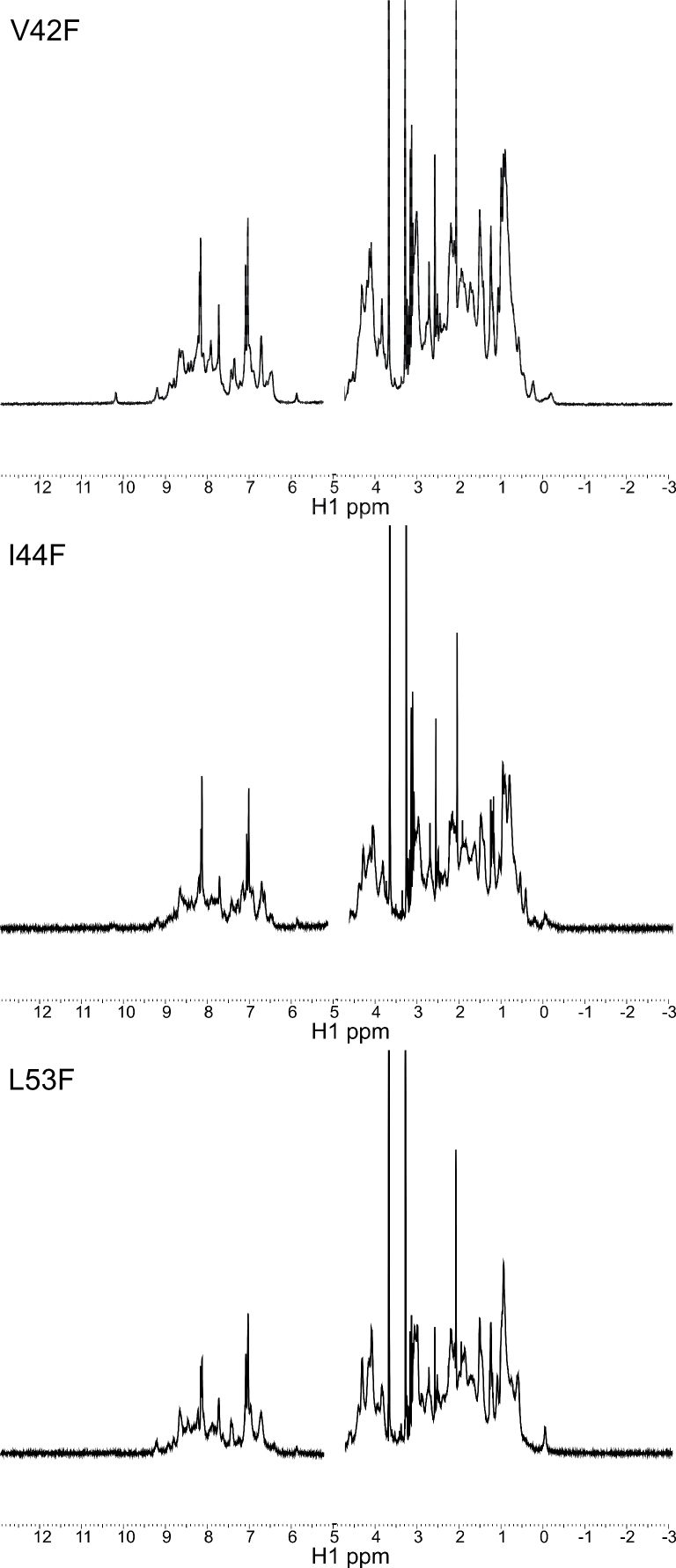
**

**SI Figure 4**

**
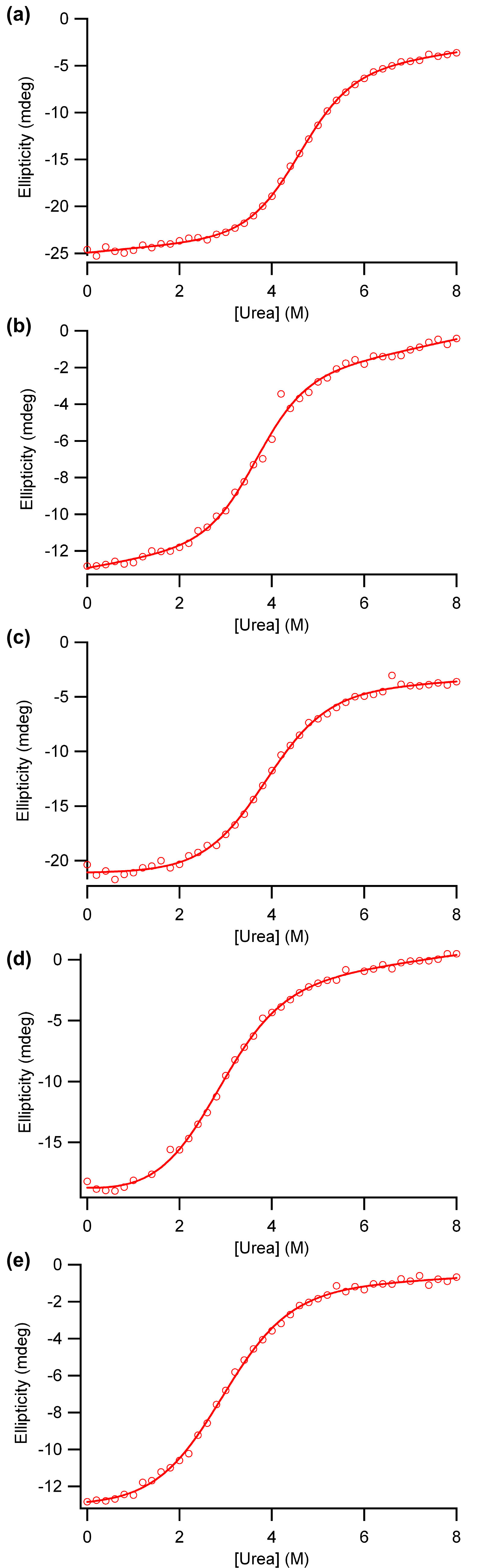
**

SI Table 1

| **Variant** | **k_UN_**  **(m_UN_)** | **k_NU_**  **(m_NU_)** | **ΔG°_UN_**  **(kJ.mol^-1^)** | **Φ_TS_** | **β_TS_** |
| --- | --- | --- | --- | --- | --- |
| L16F | 675.2 ± 25.1  (5.00 ± 0.04) | 0.11 ± 0.01  (0.30 ± 0.03) | -20.6 ± 0.2 | 0.23 ± 0.03 | 0.94 ± 0.01 |
| V68F | 986.8 ± 34.4 (5.06 ± 0.04) | 0.46 ± 0.02  (0.43 ± 0.02) | -18.0 ± 0.1 | 0.05 ± 0.01 | 0.92 ± 0.00 |
| Im9 | 1164.4 ± 41.9 (4.69 ± 0.03) | 0.02 ± 0.00  (0.45 ± 0.09) | - 26.2 ± 0.6 | - | 0.91 ± 0.02 |
